# Imaging in scrub typhus – a pictorial review

**DOI:** 10.1007/s00261-026-05474-5

**Published:** 2026-04-01

**Authors:** Shravan Reddy Kankara, Shreyas Reddy Kankara, Dhanush Jayanna, Galib Mirza Nasirul Islam, Nishant Aswani, Simmi Kumari, Yashant Aswani

**Affiliations:** 1 St.John’s Medical College Hospital, Bengaluru, India; 2 University of Iowa Health Care, Iowa City, USA; 3 Geetanjali Medical College and Hospital, Udaipur, India; 4 Jaipur National University, Jaipur, India

**Keywords:** *Orientia Tsutsuganushi*, Scrub typhus, Zoonosis, Acute febrile Illness, Rickettsial infection, Multisystem Imaging

## Abstract

Scrub typhus (STy) is a common yet underrecognized and life-threatening rickettsial zoonosis endemic to the Asia-Pacific region. It is transmitted by the bite of chigger mites infected with *Orientia tsutsugamushi*. In the US, it is primarily encountered among recent travellers. STy presents as an acute febrile illness with non-specific features. Eschar at the site of a mite bite and positive serology clinch the diagnosis. However, the classic eschar may not be seen in all cases, and serology may be negative in early cases. Multisystemic involvement is the rule in STy, and the underlying pathology is vasculitis & perivasculitis across multiple organs. Imaging features are nonspecific. However, awareness about common radiological features such as a combination of gallbladder wall edema, hepatosplenomegaly, and reticulonodular lung opacities is crucial to raise suspicion of STy in those at risk and thus prompt specific diagnostic testing. Imaging features are particularly useful in flagging cases of early disease with negative serology or those without the characteristic eschar. STy responds promptly to antirickettsial antibiotics. Delayed institution of antibiotics carries an adverse prognosis. Hence, early diagnosis is important. Further, imaging also helps with prognostication and early detection of complications.

**Supplementary Information:**

The online version contains supplementary material available at https://doi.org/10.1007/s00261-026-05474-5.

## Introduction

 Scrub typhus (STy) is an acute, potentially life-threatening zoonotic infection caused by *Orientia tsutsugamushi* (*OT*), an obligate intracellular Gram-negative bacterium [1]. It is an important cause of acute undifferentiated febrile illness in tropical and subtropical regions and remains a significant yet under-recognized contributor to infectious disease–related morbidity and mortality.

**Fig. 1:**
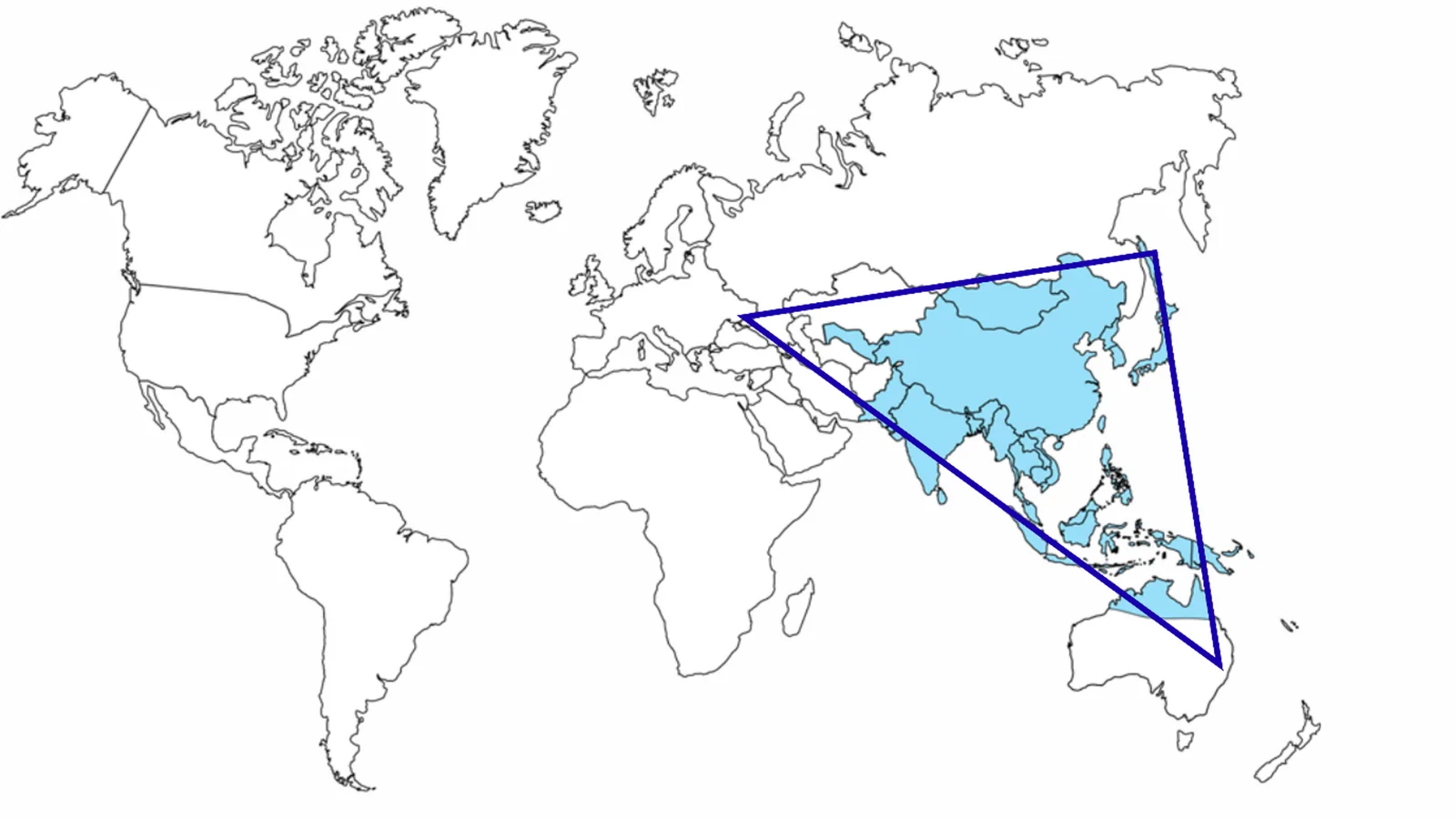
Global map illustrating the endemic distribution of scrub typhus (tsutsugamushi disease), with endemic areas shaded in blue. The disease is endemic in Eastern Asia and Western Pacific region, from Japan to India and Pakistan, and Korea to Australia (“tsutsugamushi triangle”)

The disease is classically endemic within the “tsutsugamushi triangle,” encompassing South and Southeast Asia, Northern Australia, and the Western Pacific Islands [2]. However, recent epidemiological evidence suggests a substantially wider burden than previously appreciated. A global meta-analysis has demonstrated seropositivity rates of approximately 10% in healthy populations and over 23% among febrile patients, highlighting both widespread exposure and significant underdiagnosis [3] (Fig. 1).

Despite the availability of effective antibiotic therapy, STy continues to be associated with appreciable mortality. Contemporary studies report median mortality rates of approximately 5–7% among hospitalized patients and around 2% in outpatient settings, figures that remain concerningly comparable to the previously reported untreated median mortality rate of 6% [3, 4].

The disease is most prevalent in rural areas, where access to healthcare facilities, advanced diagnostics, and timely treatment is often limited. Clinical diagnosis is frequently challenging due to the absence of a characteristic eschar in a substantial proportion of patients and the limited sensitivity of serological tests during the early phase of illness, when prompt initiation of therapy is most critical. Laboratory confirmation using reference standards may be costly or impractical in many endemic, resource-constrained settings [4].

Given the protean clinical manifestations and multisystem nature of STy, familiarity with its imaging spectrum is essential for radiologists and clinicians alike. This pictorial review illustrates the imaging findings of STy across organ systems, with emphasis on their role in early diagnosis, prognostication, and therapeutic guidance.

## Life cycle

Transmission to humans occurs through the bite of infected larval trombiculid mites, primarily of the *Leptotrombidium* genus [5]. The life cycle of this mite comprises eggs, larva (chigger), nymph, and adult in that order. Disease transmission occurs only during the larval stage, which is the only parasitic form that feeds on rodents, the natural reservoir. Humans are accidental hosts, following occupational or recreational exposure to vegetation harboring chiggers [6].

The persistence of *OT* in endemic regions is maintained through both transovarial (vertical) and transstadial (across life stages) transmission within the mite population [7]. STy demonstrates seasonality, with incidence typically peaking during the rainy and post-monsoon seasons. However, the seasonal pattern may vary by geographic region, with some areas reporting cases throughout the year [8].

## Pathogenesis

After inoculation, *OT* multiplies locally and disseminates systemically, primarily targeting endothelial cells lining the blood vessels across multiple organs, including the heart, lungs, brain, liver, kidneys, pancreas, skin, and spleen. This widespread endothelial infection leads to vasculitis and perivasculitis, the hallmarks of STy [9].

**Fig. 2:**
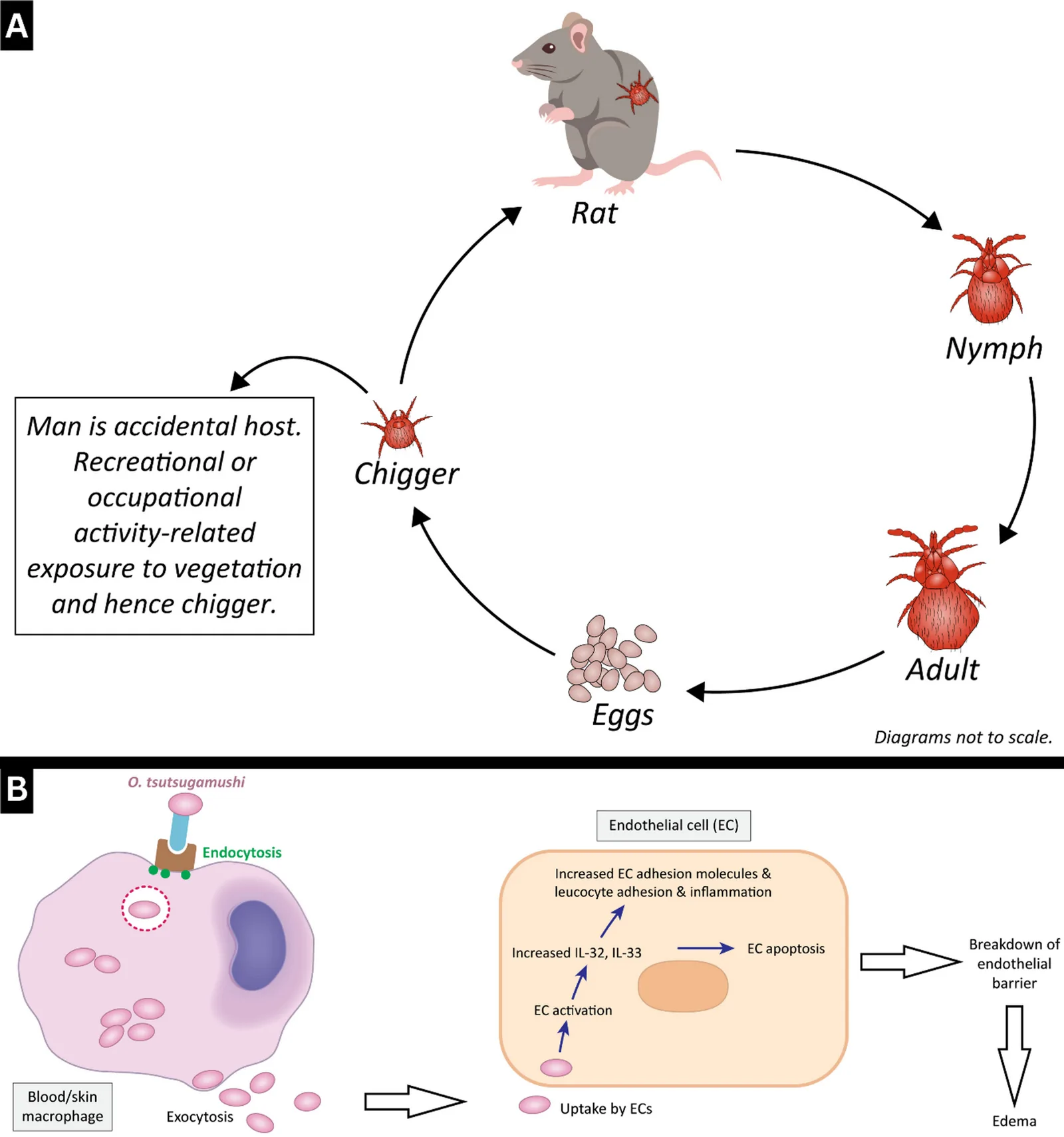
**A** Life cycle of trombiculid mites (Leptotrombidium spp.). The larva (chigger) feeds only once on a vertebrate host, transmitting *Orientia tsutsugamushi*. The bacterium is maintained from one generation of mite to another by transovarial transmission. **B** Diagrammatic overview of scrub typhus pathogenesis, highlighting endothelial invasion and downstream vascular injury

The host immune response involves both humoral and cell-mediated mechanisms, with elevated circulating cytokine levels during the acute phase of infection [10]. Activation of cytotoxic T lymphocytes and natural killer cells contributes to pathogen clearance through the destruction of infected endothelial cells [11]. However, *OT* employs immune evasion strategies that enable persistence within the vascular endothelium. Notably, downregulation of glycoprotein 96 in infected cells impairs antigen presentation via major histocompatibility complex class I pathways, reducing effective immune recognition [12] (Fig. 2).

## Clinical manifestations

Despite its significant burden, STy remains underdiagnosed and underreported, largely due to its nonspecific clinical presentations, which overlap with other febrile illnesses such as malaria, dengue, leptospirosis, and typhoid fever [1]. STy typically presents as an acute undifferentiated febrile illness, with an incubation period of 6–21 days [13]. Common symptoms include abrupt-onset high-grade fever with chills, headache, myalgia, nausea, vomiting, and abdominal discomfort [14]. A maculopapular rash may develop in the second week of illness, usually beginning on the trunk and spreading to the extremities [1].

**Fig. 3:**
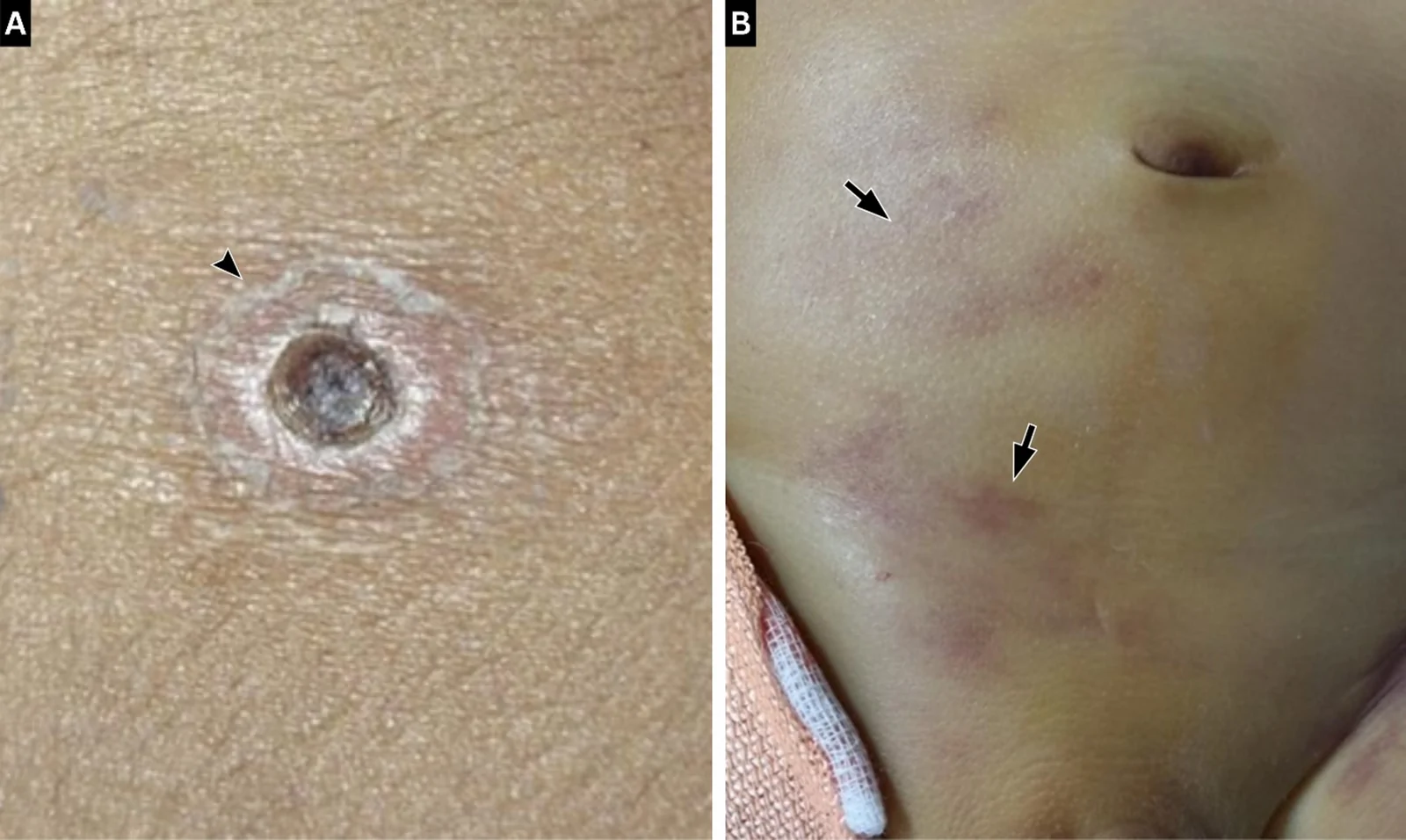
**A** Clinical photograph shows an eschar over the right upper back (arrowhead), characterized by a central black scab with surrounding erythema, in a 45-year-old man who presented with 4 days of fever and myalgia. **B** Clinical photograph shows a purpuric rash (solid black arrows) in a 4-year-old male with fever, abdominal distension, myalgia, and disseminated intravascular coagulation

A characteristic finding is an eschar, a necrotic, non-itchy, painless lesion with a black crust and surrounding erythema, typically located at the site of the chigger bite. It begins as a small papule, which gradually enlarges and undergoes central necrosis to form the classic black scab [15]. Eschars are often found in areas of skin friction, such as the axilla, groin, waist, and neck [1, 8]. However, its detection can be variable and is influenced by geographic, ethnic, and observer-related factors [8, 16] (Fig. 3).

Key laboratory findings in STy include thrombocytopenia, elevated liver enzymes, and increased procalcitonin levels [17].

## Diagnosis

Isolation of *OT* is the diagnostic gold standard but is limited by prolonged turnaround time (~28 days) and the requirement for Biosafety Level- 3 facilities. As an obligate intracellular pathogen, it cannot be cultured in standard media and requires cell lines such as L929, Vero, or HeLa. Shell vial culture has been proposed to reduce culture time to 48–72 h [18].

Polymerase chain reaction (PCR) is the most effective method for early diagnosis of STy, as it detects *OT* DNA in blood or eschar samples before seroconversion [19, 20]. Quantitative PCR assays, particularly those targeting the 47-kDa gene, demonstrate high diagnostic accuracy, with reported sensitivities of approximately 97–98% and specificity approaching 100% [21].

The indirect immunofluorescence assay (IFA) remains the serological reference standard. However, reported IgM diagnostic cutoff titers vary widely, ranging from 1:10 to 1:25,600, with substantial inter- and intraregional variability and limited region-specific validation [22]. This variability results in inconsistent specificity, reported between 74% and 99.7% across studies [21].

IgM ELISA shows good diagnostic performance, though optimal optical density (OD) cut-off values vary by region and assay. Reported OD cut-offs range from 0.5 to 1.25 [23], with sensitivities of approximately 92–93% and specificities of 91–92% [21, 24]. False-positive results occur in about 8% of cases due to IgM cross-reactivity with other acute febrile illnesses, including leptospirosis, tuberculosis, malaria, and enteric fever [21].

Rapid diagnostic tests (RDTs) for STy provide a valuable point-of-care diagnostic option, particularly in peripheral and resource-limited settings. Among available assays, the ImmuneMed Scrub Typhus Rapid RDT has demonstrated superior performance, with reported sensitivity and specificity of 98.6% and 97.6%, respectively, compared with SD Bioline (84.4% sensitivity, 96.3% specificity) [21, 25]. A meta-analysis demonstrated substantial inter-study variability in diagnostic accuracy, even among RDTs from the same manufacturer [26].

The traditional Weil–Felix test, based on cross-reactive Proteus antigens, has limited diagnostic utility due to poor and highly variable sensitivity despite generally good specificity. Reported sensitivities range from as low as ~30% at a cutoff ≥ 1:80 [27], while higher cutoffs (≥ 1:160) improve specificity to ~94% but yield only modest sensitivities (~40–67%) [28, 29]. Consequently, the test is largely restricted to preliminary screening in resource-limited rural settings.

## Imaging findings

Clinical and imaging manifestations categorized by organ system are summarized in Table 1.

**Table 1:** Overview of the clinical and imaging manifestations of scrub typhus

SYSTEM	MANIFESTATIONS
Pulmonary	Pleural effusion (42–68%), ground-glass opacities (21–40%), consolidation (10–25%), interlobular septal thickening (10–47%), centrilobular nodules (~11–47%), pulmonary edema (~14–16%), ARDS (6–14%), pulmonary hemorrhage (rare).
Gastrointestinal and Abdominal	
Liver	Hepatomegaly (~74%), periportal edema (28–54%), inhomogeneous hepatic enhancement (34–47%).
Abdominopelvic lymph nodes	Multistation lymphadenopathy (~47–87%).
Spleen	Splenomegaly (47–85%), splenic infarcts (4–16%), spontaneous splenic rupture (rare).
Gallbladder	Diffuse gallbladder wall thickening with edema (17–47%), acute acalculous cholecystitis (rare).
Kidney	Acute kidney injury (13–53%); enlarged kidneys with increased cortical echogenicity on US; bilateral renal enlargement and persistent/striated nephrogram on CT.
Pancreas	Acute pancreatitis (uncommon).
Gastrointestinal tract	Gastrointestinal bleeding (hematemesis, melena, hematochezia) (6–9%).
Cardiovascular	Myocarditis (~4.3%), pericarditis, pericardial effusion, acute heart failure, arrhythmias.
Central Nervous System and Spine (25–46%)	Meningoencephalitis, vasculitic infarcts (DWI restriction), intracranial hemorrhage (SWI/GRE), ADEM-like lesions, cranial nerve enhancement (VI, VII), cerebellitis, LETM
Peripheral Nervous System	Guillain–Barré syndrome (nerve root enhancement), Miller Fisher syndrome (rare).

## Role of radiology in scrub typhus

Imaging in STy is important to identify the extent of involvement in at-risk patients. Further, imaging helps in raising suspicion for cases where serology is unyielding and or the characteristic eschar is missing. Imaging in STy should be tailored to the predominant clinical presentation and severity of organ involvement. Ultrasound (US) is generally the first-line modality for evaluation of abdominal symptoms, particularly in endemic rural regions, as US is widely available and relatively inexpensive. US can be used to monitor the progression of the gallbladder wall edema, a common feature of STy. CT is the workhorse for thoracic and neurological evaluation, particularly for acute, emergent evaluation. However, MRI, owing to its superior soft tissue contrast resolution, is the modality of choice for interrogation of intracranial findings. For e.g., diffusion-weighted imaging helps identify vasculitic infarcts in the brain [30–32].

## Pulmonary manifestations

Pulmonary manifestations range from mild pneumonitis to life-threatening acute respiratory distress syndrome with type I respiratory failure [33]. Other manifestations of STy include pneumonia, pleural effusion, pulmonary edema, and pulmonary hemorrhage [30, 34].

Overall, radiographic abnormalities have been documented in 51–72% of patients [16, 35]. The most frequent chest radiograph findings include pleural effusions (~14–43%) [35, 36], reticulonodular opacities (~10–41%) [35, 36], airspace consolidation (~10–25%) [16, 35], and peribronchial infiltrates (~5–21%) [35, 36], often with bilateral involvement and a lower lung zone predominance [16, 36]. Pulmonary edema and congestion are reported in approximately 14–16% of cases [36], while acute respiratory distress syndrome is less common (~6–14%) [35, 36]. In a minority of patients, pleural effusion may represent the sole radiographic abnormality. Notably, a large systematic review reported a case fatality rate of 26.8% among patients with ARDS, underscoring the clinical severity of pulmonary involvement [37].

**Fig. 4:**
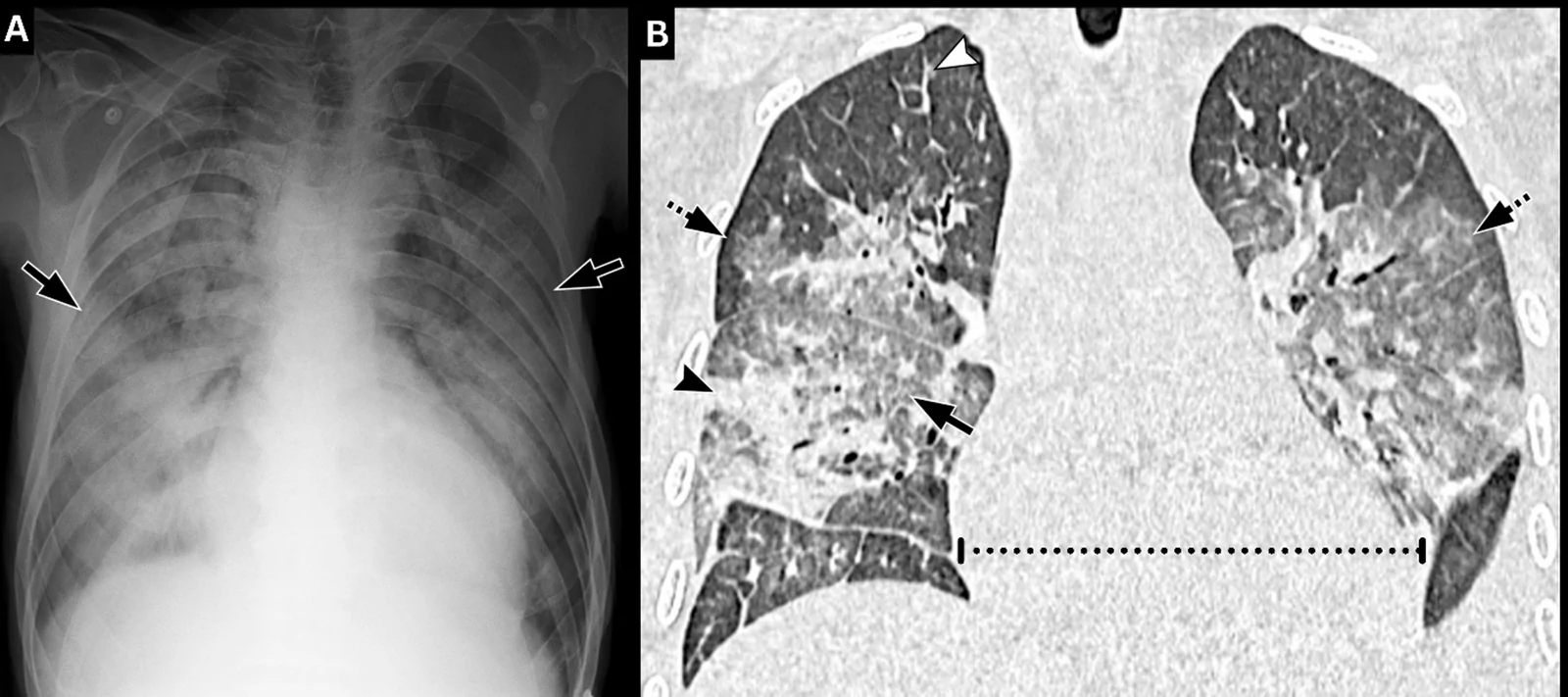
Pulmonary manifestations in scrub typhus. **A **Chest radiograph in a 70-year-old male with 4 days of fever and 1 day of dyspnea shows diffuse bilateral consolidation (solid black arrows), consistent with pneumonia. He was treated with IV doxycycline for 8 days & discharged 6 days later. **B **Coronal HRCT chest in a 20-year-old female with fever and dyspnea demonstrates bilateral perihilar ground-glass opacities (dotted black arrows) with patchy consolidation (black arrowhead), smooth interlobular septal thickening (solid black arrow), and cardiomegaly (black dotted line), consistent with interstitial pulmonary edema

Computed tomography (CT) of the thorax is more sensitive than chest radiography in detecting pulmonary involvement. Pleural effusion is the most frequent CT finding, reported in approximately 42–68% of cases [38, 39], and may be the only abnormality in a subset of patients [39]. Ground-glass opacities are observed in approximately 21–40% of patients [38, 39], while consolidation is reported in approximately 10–25% [38, 39], with bilateral involvement being more common than unilateral disease [39]. Interlobular septal thickening is reported in approximately 10–47% [16, 39], and centrilobular nodules are noted in ~11–47% [16, 30]. Contrast-enhanced CT frequently demonstrates enlarged, non-necrotic mediastinal, hilar, and axillary lymph nodes [30] (Fig. 4).

## Abdominal manifestations

### Liver

Liver involvement is common in STy, reported in approximately 75–92% of patients [36]. Jaundice may be observed, and in severe cases can progress to acute liver failure, defined by encephalopathy and/or jaundice with markedly elevated liver enzymes [40].

Biochemical abnormalities typically demonstrate a hepatocellular pattern, with elevated aspartate aminotransferase (AST/SGOT) in approximately 78–80% of patients [41, 42] and alanine aminotransferase (ALT/SGPT) in approximately 63–70% [41, 42]. In contrast, alkaline phosphatase elevation is less frequent (~27–50%) [41, 42], while hyperbilirubinemia is reported in approximately 16–62% of cases [41, 42]. The predominance of aminotransferase elevation over cholestatic markers correlates with sinusoidal endothelial vasculitis and focal hepatocellular injury [42]. Autopsy studies further demonstrate submassive hepatic necrosis, lymphocytic infiltration of the hepatic capsule, and widespread fibrin thrombi within hepatic sinusoids [40].

**Fig. 5:**
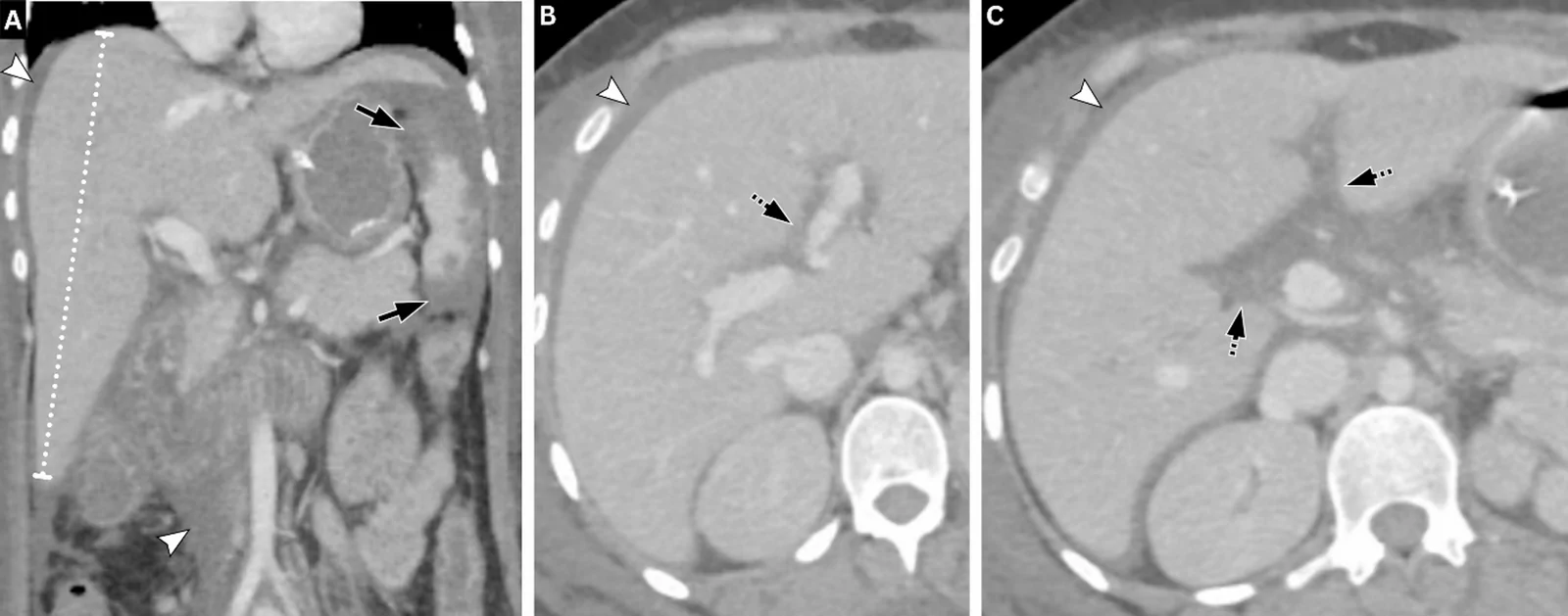
Hepatic involvement in scrub typhus in a 31-year-old female who developed diffuse cerebral edema, acute respiratory distress syndrome, generalized lymphadenopathy, necrotizing fasciitis, and myositis. **A** Coronal CECT image demonstrates hepatomegaly (white dotted line), multiple hypoenhancing wedge-shaped splenic infarcts (solid black arrows), and small volume ascites (white arrowhead). **B**, **C** Axial CECT images additionally demonstrate periportal edema (black dotted arrows) and small-volume ascites (white arrowhead)

On imaging, US may demonstrate hepatomegaly with increased hepatic echogenicity [43]. Contrast-enhanced CT commonly shows hepatomegaly, reported in approximately 74% of patients [31, 44]. Additional CT findings include inhomogeneous hepatic enhancement, observed in approximately 34–47% of cases [30, 31], and periportal areas of low attenuation or periportal edema, reported in approximately 28–54% [31, 42] (Fig. 5). These imaging findings reflect hepatic congestion and periportal edema secondary to increased vascular permeability, which may result in obstruction of hepatic venous outflow and compression of the portal vein with dilation of the peribiliary plexus. Compensatory hepatic arterial hyperperfusion may contribute to the heterogeneous enhancement pattern observed on CT [31]. However, these findings are nonspecific and may mimic other hepatic conditions such as liver cirrhosis, acute cholecystitis, acute cholangitis, or liver abscess [31].

### Abdominopelvic lymphadenopathy

STy commonly demonstrates abdominopelvic lymphadenopathy, initially involving regional draining nodes near the chigger bite site, with generalized, non-necrotic lymphadenopathy developing later. On CT, abdominopelvic lymphadenopathy has been reported in approximately 47–87% of patients across published series [30, 31].

In a large CT-based series, multiple nodal stations were frequently involved, with multistation abdominopelvic lymphadenopathy reported in approximately 72% of cases. In this cohort, the periportal region was the most commonly affected site, with periportal lymphadenopathy observed in approximately 55% of patients. Extraperitoneal lymphadenopathy involving the upper and lower retroperitoneum and pelvic regions was also common, reported in approximately 73% of cases. Additional involved sites included the perigastric, para-aortic, inguinal regions, and the splenic hilum [31].

**Fig. 6:**
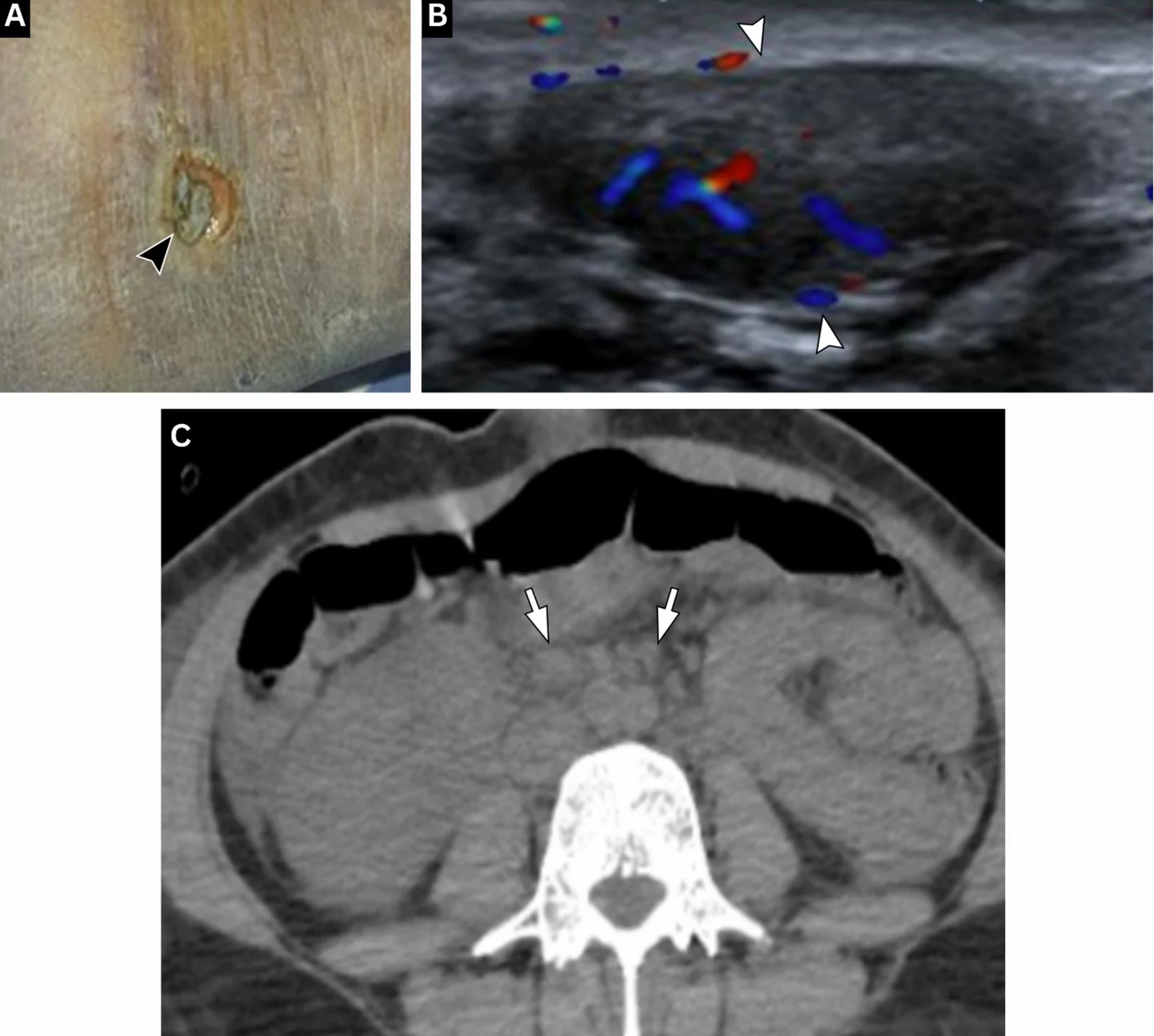
**A**, **B** Clinical photograph shows an eschar over the left heel (black arrowhead, A) in a 45-year-old male who presented with fever and myalgia since 4 days. **B** Ultrasound demonstrates an enlarged left inguinal lymph node (white arrowheads) with preserved fatty hilum (regional lymphadenopathy). **C** Noncontrast CT shows mesenteric lymphadenopathy (solid white arrows) in a 29-year-old male who presented with pneumonia and acute kidney injury, which was subsequently attributed to scrub typhus

Although periportal lymphadenopathy can also occur in acute hepatitis, STy more often shows a diffuse, multistation pattern extending beyond the periportal region to involve perigastric and extraperitoneal nodes. When seen in combination with periportal edema, hepatosplenomegaly, and a clinically apparent eschar, this is suggestive of STy. Ipsilateral pelvic lymphadenopathy may further suggest the site of an occult lower-extremity eschar (Fig. 6). Among CT findings, perigastric lymphadenopathy has been reported to correlate with hepatic dysfunction and may prompt careful evaluation of liver function [31].

### Spleen

**Fig. 7:**
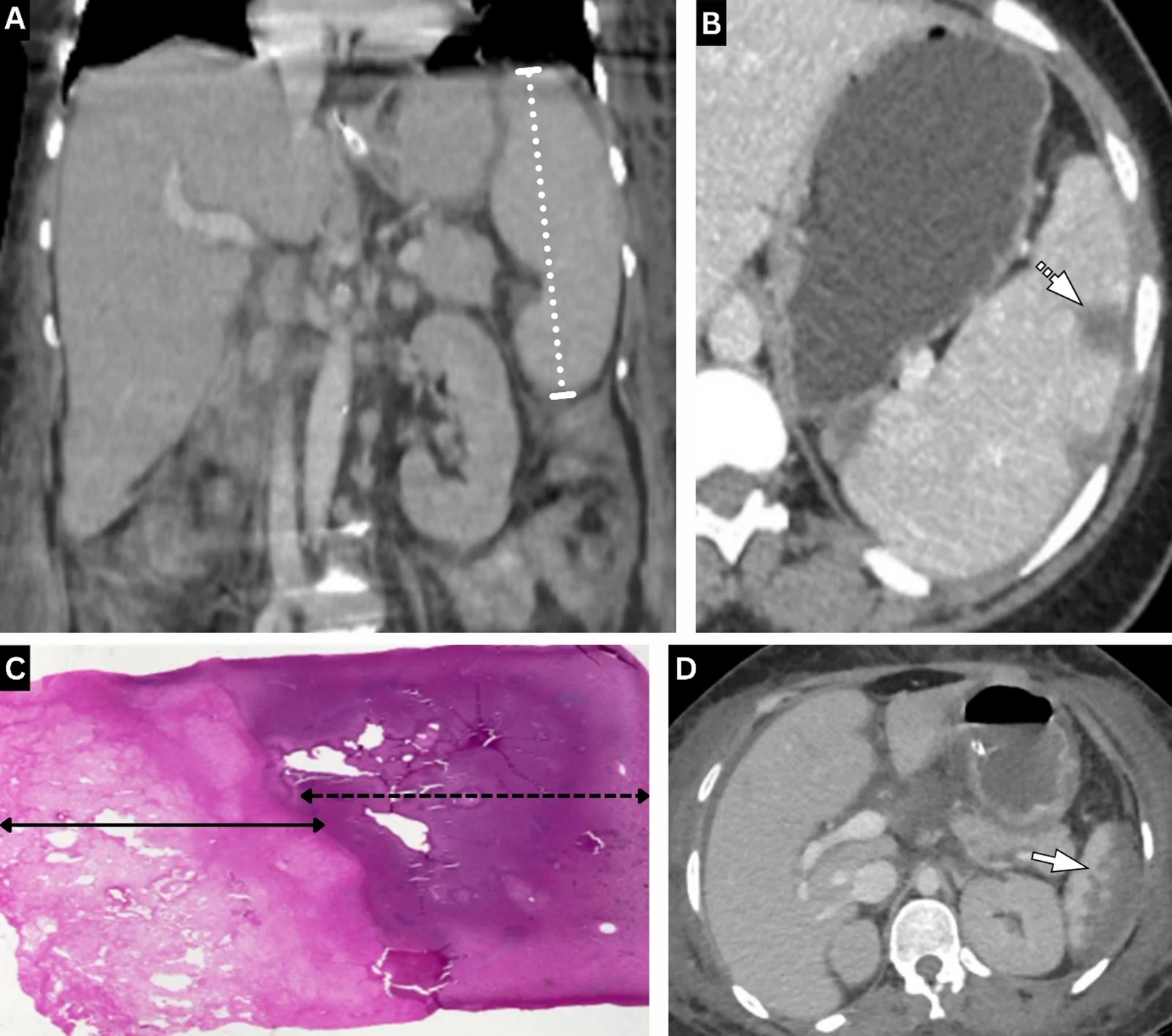
Splenic involvement in scrub typhus. **A** Coronal contrast-enhanced CT (CECT) in a 58-year-old male demonstrates splenomegaly (white dotted line). **B** Axial CECT in a 25-year-old female shows hypoattenuating wedge-shaped splenic infarcts (white dotted arrow). **C** Low-power hematoxylin and eosin-stained image of the spleen in a 25-year-old male depicts pale-staining, infarcted splenic parenchyma (double-headed solid black arrow) adjacent to preserved parenchyma (double-headed dashed black arrow), with a sharp interface between the two. **D** Axial CECT in a 31-year-old female shows multiple hypoenhancing, wedge-shaped subcapsular splenic infarcts (solid white arrows)

Splenomegaly is common, typically resulting from acute splenic congestion. It has been reported in approximately 47–85% of patients across published CT-based series, although reported prevalence varies depending on the imaging plane and size criteria used for definition [31, 42]. Splenic infarcts have also been reported in approximately 4–16% [30, 31], appearing on imaging as peripheral, wedge-shaped hypodense areas [31] (Fig. 7).

**Fig. 8:**
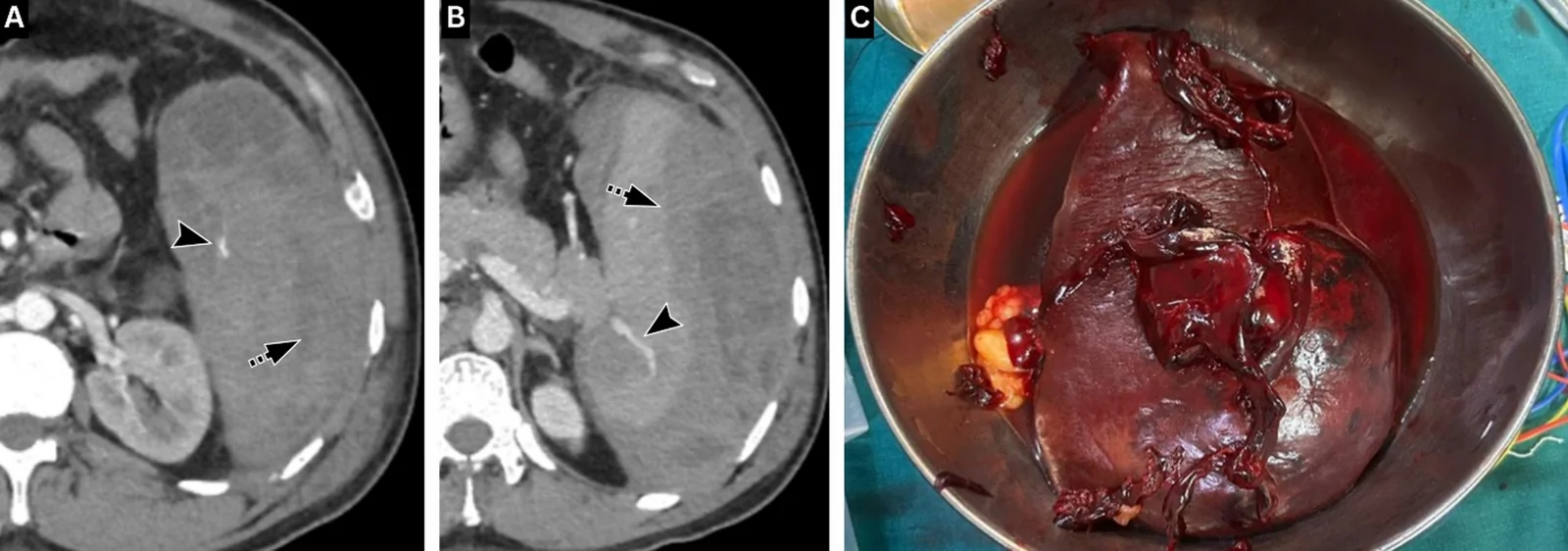
Spontaneous splenic rupture in scrub typhus in a 37-year-old male with fever, abdominal pain, and thrombocytopenia. Axial arterial (**A**) and venous (**B**) phase CT demonstrates active intrasplenic contrast extravasation, consistent with ongoing hemorrhage (black arrowhead) with subcapsular hematoma (black dotted arrows). Hemodynamic instability prompted laparotomy. **C** Gross photograph of the post-splenectomy specimen shows a ruptured spleen

A rare but serious complication is spontaneous splenic rupture leading to hemoperitoneum, described predominantly in isolated case reports. The proposed mechanism involves massive red blood cell congestion that distorts and destroys the structure of the splenic sinusoids. This, combined with splenic infarction, increases the volume of the red pulp and elevates capsular pressure, ultimately resulting in capsular distension, rupture, and extra-splenic hemorrhage [45] (Fig. 8).

### Gallbladder

**Fig. 9:**
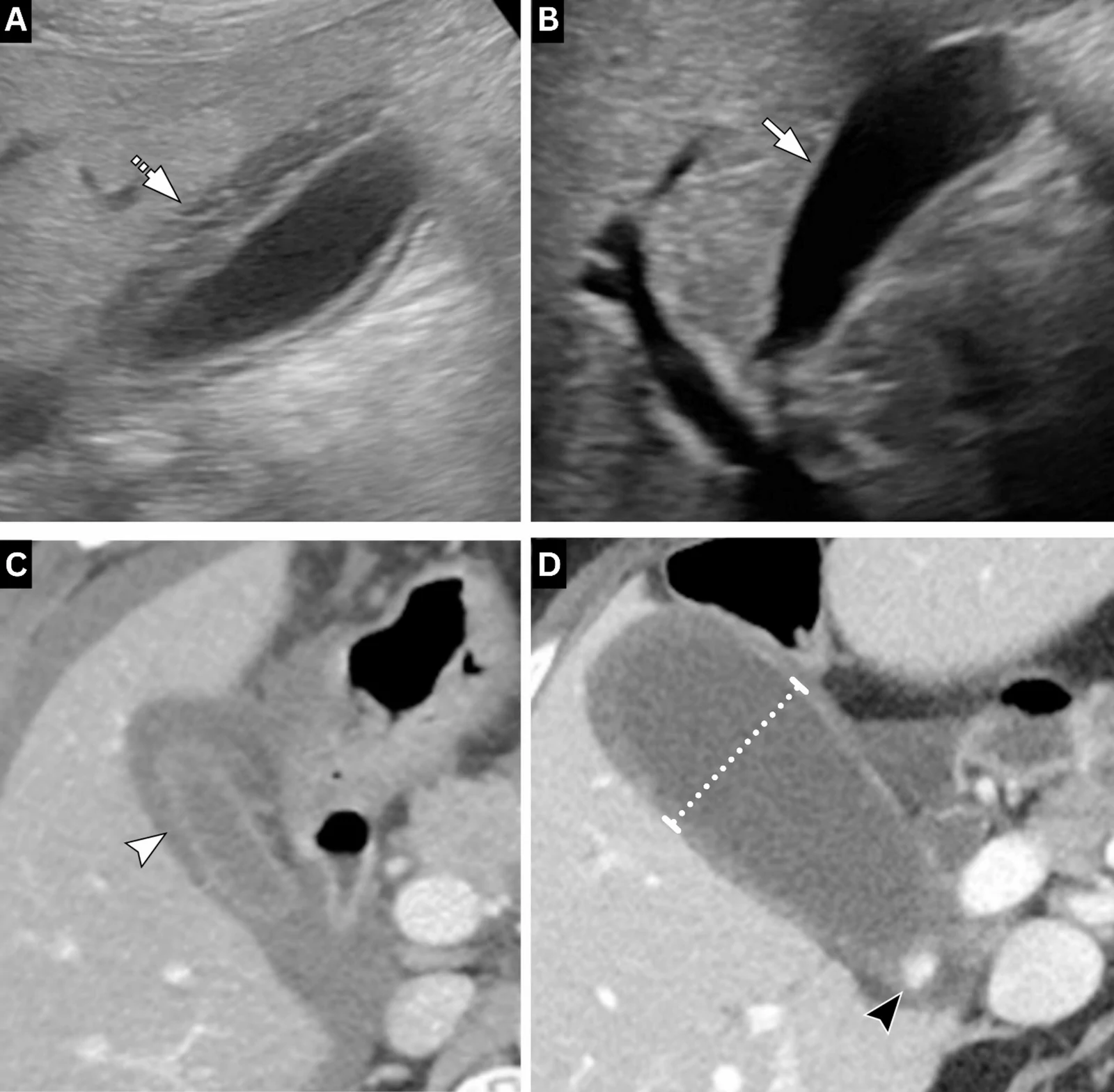
Gallbladder involvement in scrub typhus. **A**, **B** Transabdominal ultrasound shows diffuse gallbladder wall thickening with mural edema measuring up to 7 mm (white dotted arrow, A) but without tense luminal distension in a 29-year-old female, with near-complete resolution on the 3-week follow-up study (solid white arrow, B), consistent with treatment response. **C**, **D** Axial contrast-enhanced CT highlights the imaging distinction between scrub typhus-related gallbladder wall thickening and edema (white arrowhead, C) versus the tense luminal distension (dotted line, D) secondary to an obstructing gallstone in the neck (black arrowhead, D), and wall edema (measuring 4 mm) in acute calculous cholecystitis

Gallbladder wall thickening, defined as a wall thickness greater than 3 mm, is a notable imaging finding in STy, reported in approximately 17–47% of patients [30, 42]. It is often attributed to acute vasculitis and associated perivasculitis affecting the gallbladder wall [30]. This finding may mimic acute cholecystitis. However, the absence of marked gallbladder distension and the presence of diffuse subserosal edema favor scrub typhus–related involvement [30]. Rarely, STy may be complicated by acute acalculous cholecystitis, presenting with fever, right upper quadrant pain, and tenderness [46] (Fig. 9).

### Kidney

Acute kidney injury is a common yet frequently under-recognized complication of STy reported in ~13% to 53% patients and serves as an independent predictor of mortality in affected patients [47, 48]. The underlying pathophysiology is multifactorial and includes mechanisms such as multiple organ dysfunction syndrome, decreased renal perfusion from hypovolemia or shock, acute interstitial nephritis, acute tubular necrosis, rhabdomyolysis, vasculitis, and thrombotic microangiopathy secondary to disseminated intravascular coagulation [49, 50]. Urinary abnormalities are observed in 50%–80% of cases [47], with urinalysis commonly revealing oliguria, albuminuria, microscopic hematuria, and pyuria [50].

**Fig. 10:**
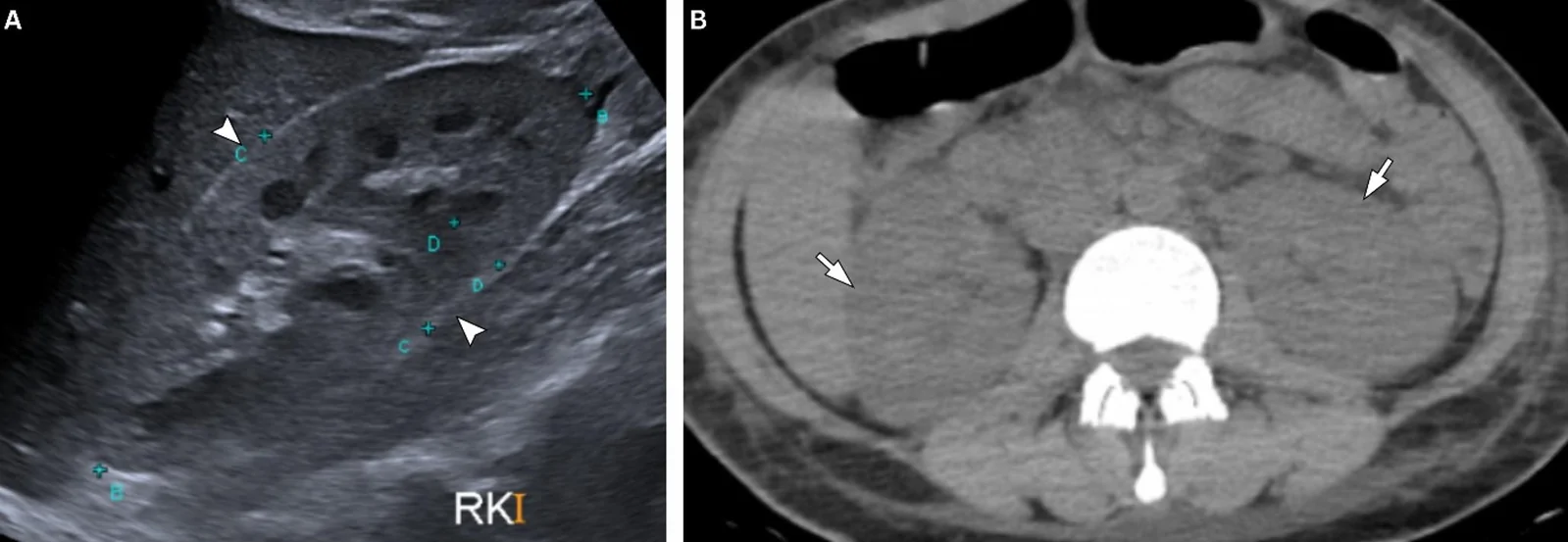
Renal involvement in scrub typhus in a 29-year-old male with rising creatinine and bilateral patchy consolidation. **A** Longitudinal transabdominal ultrasound of the right kidney demonstrates renal enlargement with increased cortical echogenicity (white arrowheads). **B** Unenhanced axial CT of the abdomen shows bilateral hypoattenuating renal parenchyma (solid white arrows), consistent with acute kidney injury

US findings are often nonspecific but may include normal-sized or enlarged kidneys with increased cortical echogenicity [51]. Contrast-enhanced CT may demonstrate bilateral renal enlargement and a persistent or striated nephrogram, indicating delayed contrast excretion and stasis within the renal tubules, findings suggestive of acute tubular necrosis or interstitial nephritis [52] (Fig. 10).

### Miscellaneous

**Fig. 11:**
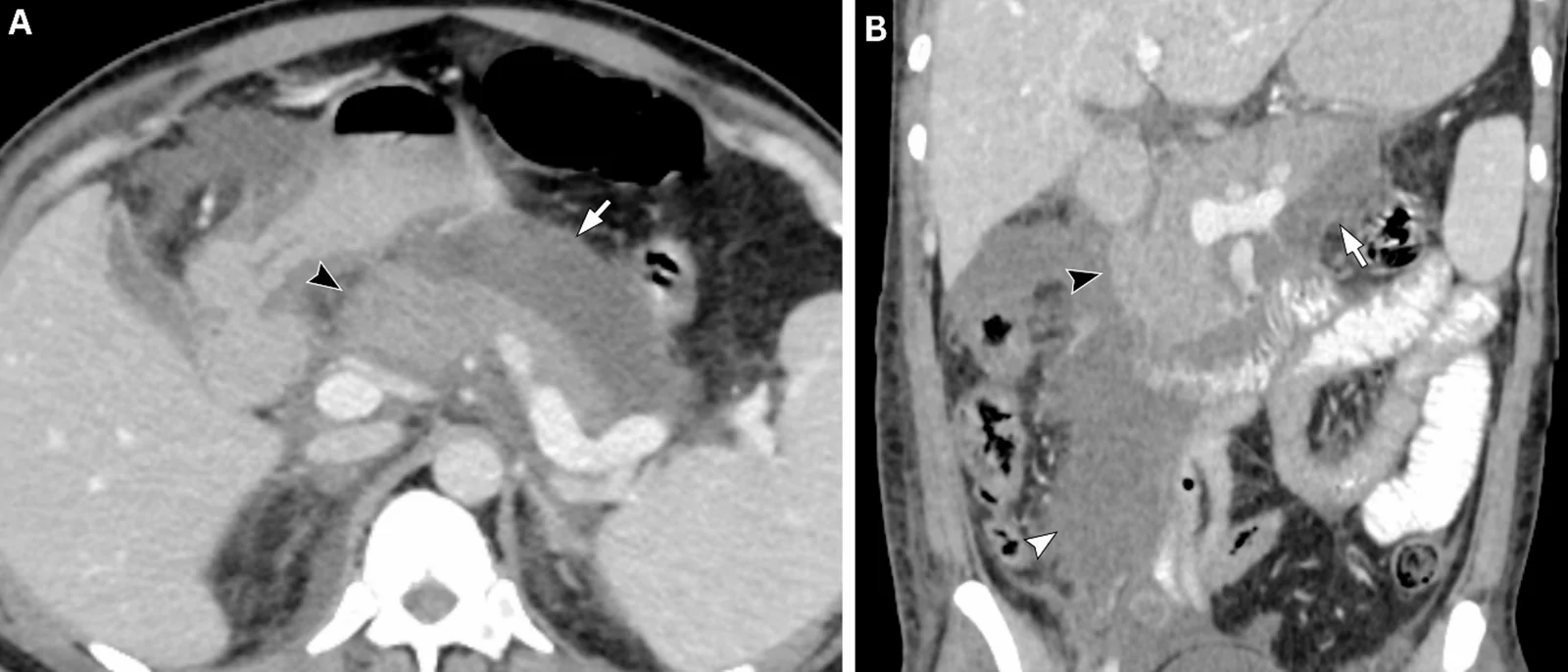
Acute interstitial edematous pancreatitis associated with scrub typhus in a 40-year-old male with serum lipase measuring 1566 U/L. **A**–**B** Axial and coronal contrast-enhanced CT (CECT) images show a mildly enlarged, edematous pancreas (black arrowhead), accompanied by acute peripancreatic fluid collection (white solid arrow) and small volume ascites (white arrowhead)

Acute pancreatitis has been reported in several cases and is considered a less frequent but established complication of the disease [53] (Fig. 11).

**Fig. 12:**
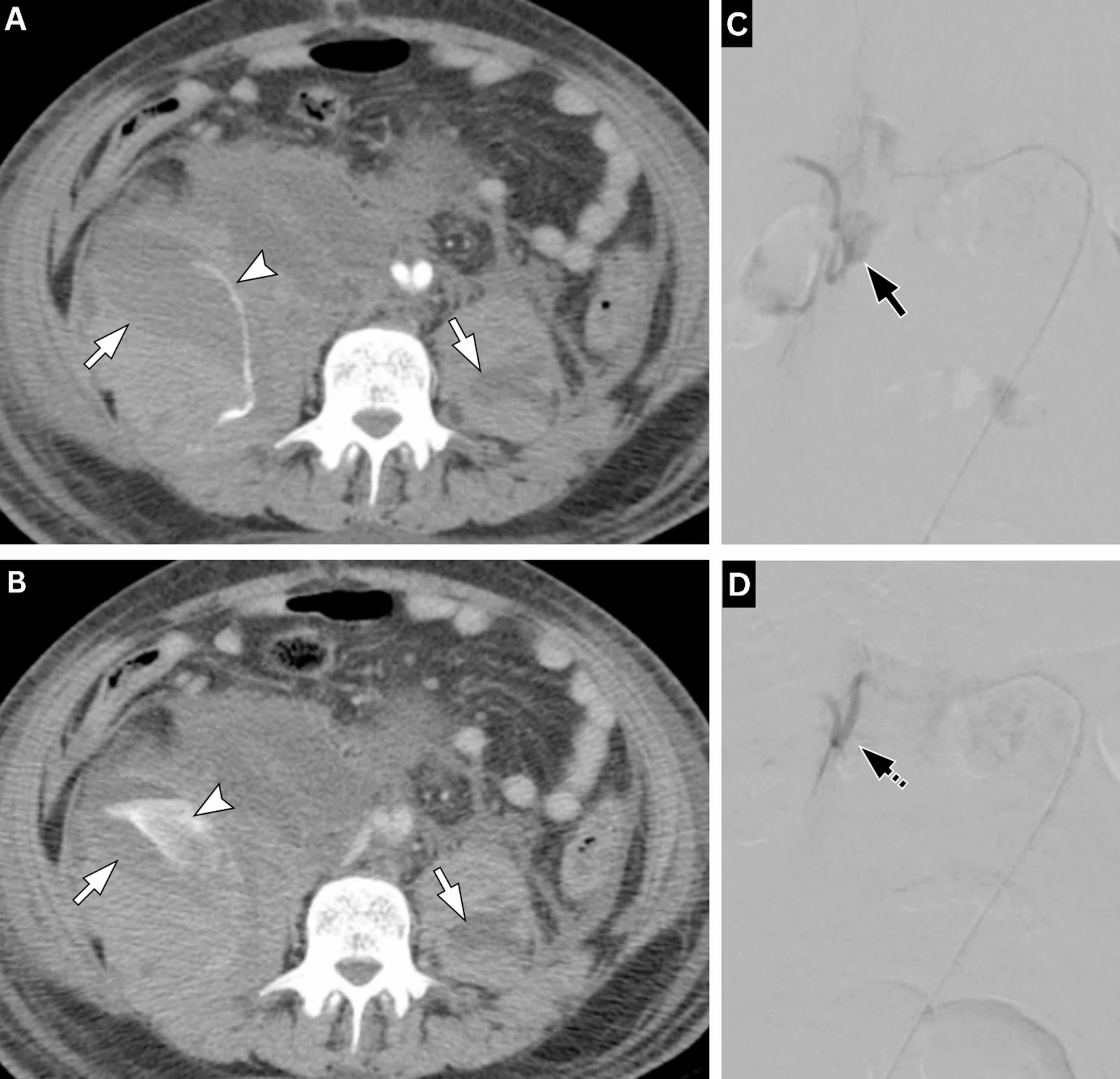
Retroperitoneal hemorrhage due to scrub typhus managed with endovascular therapy in a 52-year-old male who presented with fever, sudden-onset abdominal pain, and hypotension. Axial arterial **A** and venous **B** CT (CECT) demonstrates bilateral right greater than left psoas hematomas (solid white arrows) with active contrast extravasation on the right (white arrowheads). **C** Pre-embolization angiography confirms active contrast extravasation (solid black arrow) from the right iliolumbar artery. **D** Post-embolization angiogram shows resolution of contrast blush (black dotted arrow)

Gastrointestinal bleeding is another manifestation of STy, reported in approximately 6–9% of patients, and may present with hematemesis, melena, or hematochezia [54, 55]. Psoas hematoma is a rare hemorrhagic complication of STy. On contrast-enhanced CT, it presents as asymmetric enlargement of the psoas muscle with intramuscular hyperdense areas [56] (Fig. 12).

## CVS manifestations

Myocarditis occurs in approximately 4.3% of STy cases. Patients may present with chest pain, palpitations, fatigue, or signs of acute heart failure despite the absence of overt coronary artery disease [57]. The reported mortality rate reaches up to 24% in affected individuals [4]. Transthoracic echocardiography typically reveals a reduced left ventricular ejection fraction and, in some cases, regional wall motion abnormalities [58]. Cardiac MRI reveals myocardial edema and non-ischemic late gadolinium enhancement in subepicardial or mid-myocardial distribution [59]. Cardiac or chest CT may show delayed myocardial enhancement with iodinated contrast [60].

**Fig. 13:**
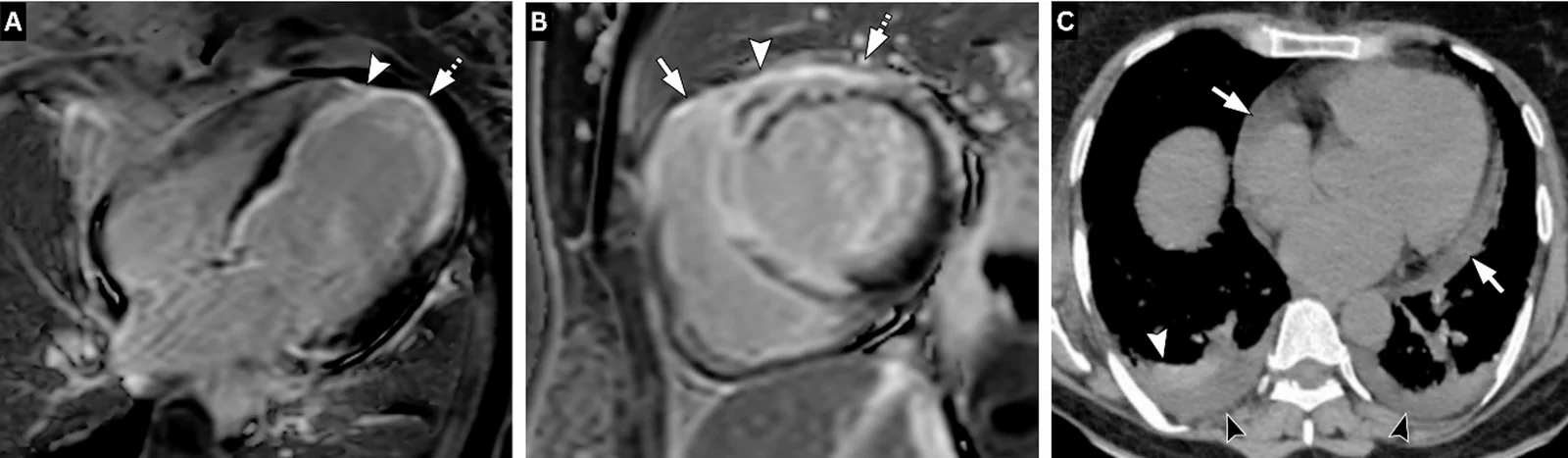
**A**, **B** Myocarditis in a 35-year-old female with scrub typhus presenting with fever, bradycardia, and lower-limb edema. Cardiac MRI four-chamber SSFP (**A**) and short-axis SSFP (**B**) demonstrate epicardial-to-mid myocardial late gadolinium enhancement of the left ventricle (white dotted arrows), with additional involvement at the right ventricle (RV) insertion points (white arrowheads) and along the RV free wall (solid white arrow). **C** Pericardial effusion in a 62-year-old female with scrub typhus and pneumonia presenting with fever and cough for 5 days and breathlessness for 1 day. Axial CT chest (**C**) shows a small volume pericardial effusion (solid white arrows). Also seen are bilateral small-volume pleural effusions (black arrowheads) and passive atelectasis of the adjacent right lower lobe (white arrowhead)

Pericarditis is a relatively common cardiac complication of STy, with an incidence as high as 58% reported in autopsy series [61]. If left untreated, it can progress to pericardial effusion and eventually cardiac failure [62] (Fig. 13).

## CNS manifestations

Central nervous system (CNS) involvement in STy can be categorized as direct invasion (meningitis, meningoencephalitis, and encephalitis), vasculitis (infarction, microhemorrhages, and venous sinus thrombosis), immune-mediated (cerebellitis, myelitis, acute disseminated encephalomyelitis, peripheral neuropathy, cranial nerve palsies, Guillain–Barré syndrome, brachial plexopathy, and polyneuropathy), and encephalopathy. Reported rates of neurological involvement in STy range from 25% to 46% [32]. Common neurological symptoms include altered sensorium, seizures, and focal neurological deficits [63–65]. Cranial neuropathies, most frequently affecting the sixth and seventh cranial nerves, may result in diplopia and facial weakness [66, 67].

Cerebrospinal fluid (CSF) analysis usually reveals mild to moderate pleocytosis, elevated protein levels, and normal glucose concentrations [68]. In certain cases, CSF profiles may mimic tuberculous or viral meningoencephalitis [69].

**Fig. 14:**
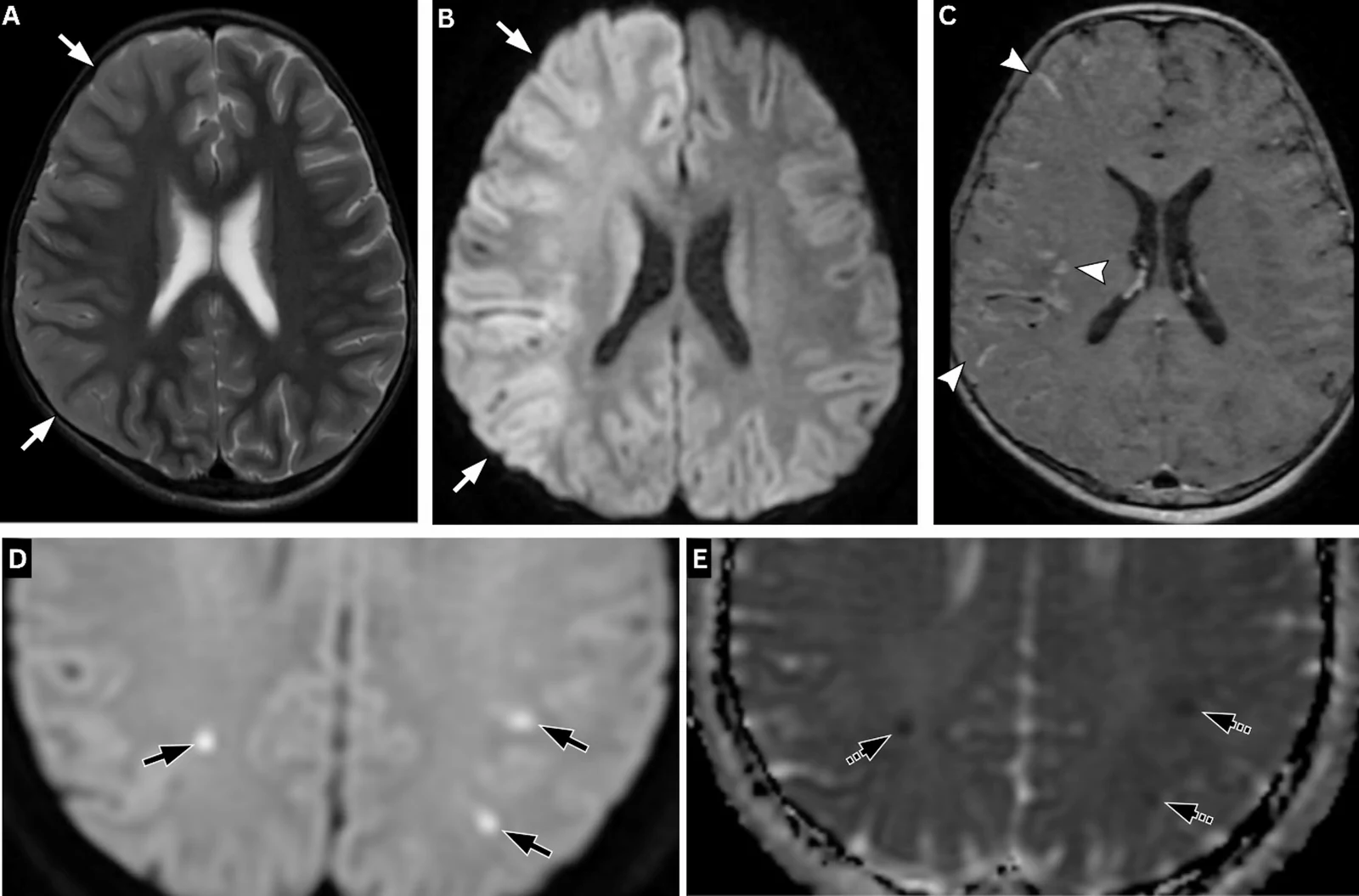
Meningoencephalitis (**A–C**) secondary to scrub typhus in a 5-year-old male with 5 days of fever and seizures. Axial T2-weighted image (**A**) and DWI (**B**) show gyral thickening with increased signal in the right fronto-parietal region (solid white arrows) with corresponding decreased signal on ADC map (not shown). Axial T1 fat-suppressed post-contrast (**C**) demonstrates leptomeningeal enhancement in the same region (white arrowheads). Vasculitis (**D**, **E**) in scrub typhus in a 49-year-old female with fever and altered sensorium for 3 days. Axial DWI (**D**) and ADC (**E**) show multiple punctate foci of diffusion hyperintensity (solid black arrows) with corresponding ADC signal drop (white arrows), consistent with vasculitic infarcts.

Neuroimaging findings in STy are nonspecific and reflect its varied neurological manifestations. In patients with meningoencephalitis or encephalopathy, MRI typically shows T2/FLAIR hyperintensities in the subcortical white matter, bilateral thalami, basal ganglia, brainstem, and cerebellar peduncles, often accompanied by leptomeningeal enhancement [70, 71]. Cerebral infarcts, often secondary to vasculitis, appear as areas of restricted diffusion on DWI, whereas intracranial hemorrhages are seen as blooming hypointense foci on SWI or GRE sequences (Fig. 14).

**Fig. 15:**
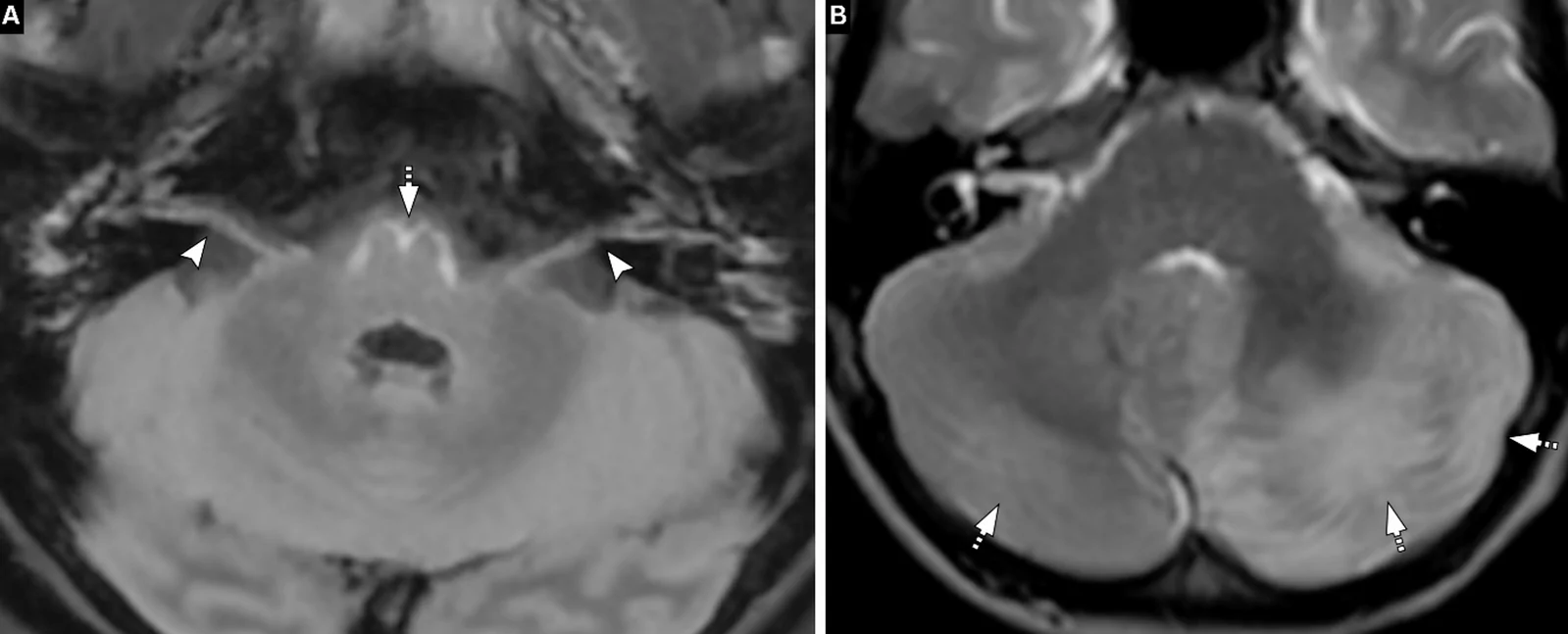
Meningitis and cranial nerve involvement. **A** A 45-year-old female with fever for 11 days, vomiting for 10 days, and bilateral lower-limb weakness for 2 days, diagnosed with scrub typhus, underwent an MRI of the brain. Axial post-contrast T2 FLAIR shows leptomeningeal thickening with enhancement along the brainstem (dotted white arrow), consistent with meningitis. Additionally, there is thickening and enhancement of bilateral vestibulocochlear nerve complexes (white arrowheads). Cerebellitis. **B** A 10-year-old female with fever, altered sensorium, and swaying, diagnosed with scrub typhus, underwent an MRI of the brain. Axial T2-weighted image shows increased signal in bilateral, left greater than right cerebellar foliae (dotted white arrows), consistent with cerebellitis

Acute disseminated encephalomyelitis (ADEM)-like changes are described as large, bilateral, ill-defined lesions involving both gray and white matter [72]. Cranial nerve involvement, particularly of the facial (VII) and abducens (VI) nerves, may be visualized as nerve thickening and post-contrast enhancement [66, 67]. Cerebellitis is characterized by T2/FLAIR hyperintensities in the cerebellar hemispheres [73] (Fig. 15). Guillain–Barré syndrome may demonstrate thickening and enhancement of the cauda equina or cranial nerve roots on contrast-enhanced spine MRI [74].

## PET-CT in scrub typhus

18 F-fluorodeoxyglucose (FDG) PET/CT has been used in STy to evaluate systemic inflammation, as activated neutrophils and monocytes/macrophages demonstrate increased glucose transporters [75]. Characteristic features include intense FDG uptake at eschar sites, generalized symmetrical FDG-avid lymphadenopathy, and diffuse splenic hypermetabolism, often with hepatosplenomegaly [76]. FDG-avid lymph nodes are typically mild to moderately enlarged, symmetrically distributed, and usually measure less than 2 cm in short-axis diameter that favors inflammatory rather than malignant involvement [75].

In the largest FDG-PET/CT series, metabolic activity in eschars, lymph nodes, and spleen showed a marked reduction following appropriate antibiotic therapy, paralleling clinical improvement. Splenic FDG uptake frequently exceeded hepatic uptake, while hepatic FDG metabolism remained largely unchanged despite hepatomegaly [76].

FDG-PET/CT may be particularly useful in eschar-negative STy and in patients with fever of unknown origin, where it can demonstrate characteristic lymphatic and splenic involvement [75]. However, FDG-avid lymphadenopathy may mimic lymphoma or metastatic disease, representing an important diagnostic pitfall, especially in patients with underlying malignancy [77].

## Hemophagocytic lymphohistiocytosis in scrub typhus

Hemophagocytic lymphohistiocytosis (HLH) is a rare, non-malignant but potentially fatal disorder of immune dysregulation that can involve multiple organ systems, secondary forms of which result from underlying infections, tumors, or autoimmune disorders. Infections may trigger excessive immune activation, leading to uncontrolled hyperactivity of natural killer cells, cytotoxic T lymphocytes, and macrophages, releasing large quantities of pro-inflammatory cytokines, resulting in a cytokine storm that culminates in tissue injury and multiorgan dysfunction [78].

**Fig. 16:**
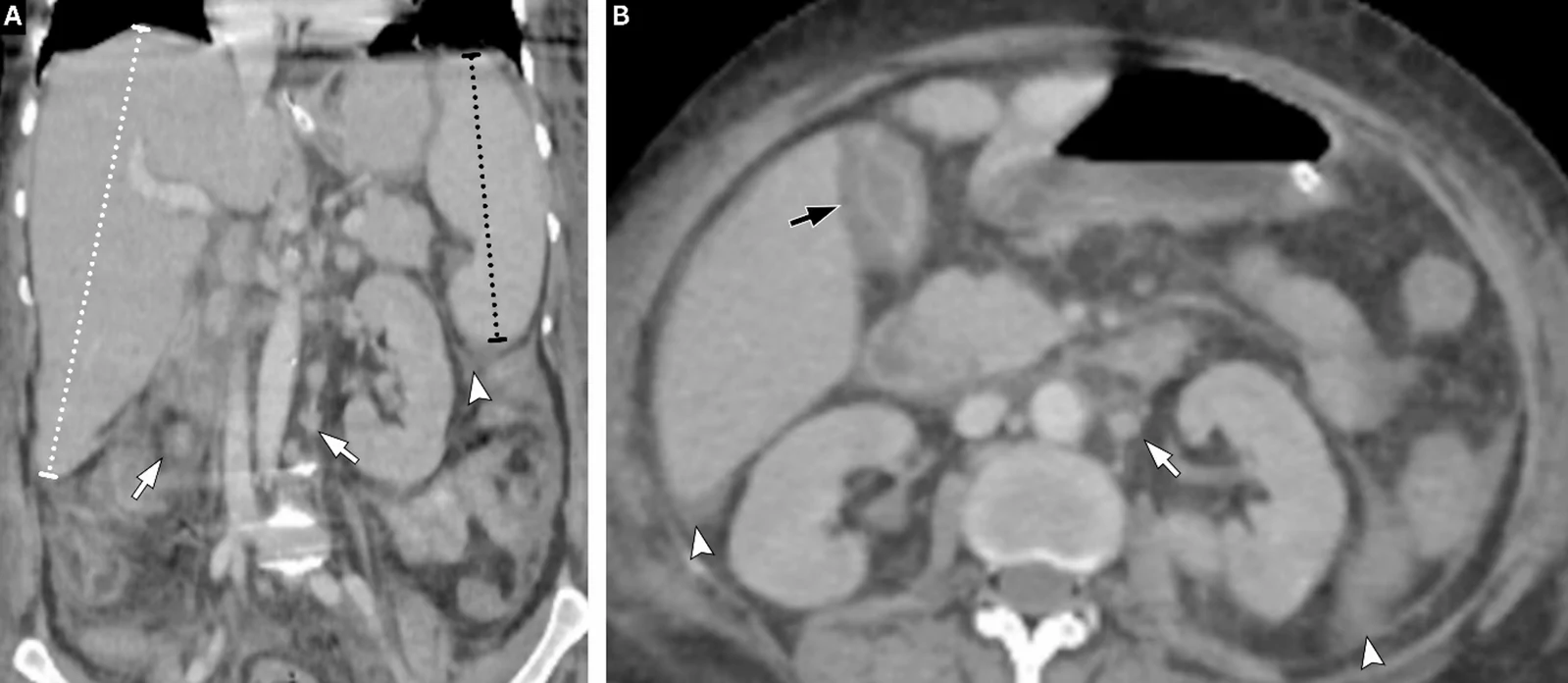
Secondary hemophagocytic lymphohistiocytosis (HLH) due to scrub typhus in a 58-year-old male. Coronal (**A**) and axial (**B**) contrast-enhanced CT images demonstrate hepatomegaly (white dotted line), splenomegaly (black dotted line), mesenteric and para-aortic lymphadenopathy (solid white arrows), gallbladder wall edema (solid black arrow), with trace ascites (white arrowheads). Laboratory workup showed elevated liver enzymes, neutropenia, thrombocytopenia, hyperferritinemia, and hypofibrinogenemia fulfilling criteria for secondary HLH

HLH associated with STy demonstrates heterogeneous and largely nonspecific manifestations, most commonly affecting the lungs, liver, spleen, central nervous system, kidneys, and hematopoietic system. Pulmonary involvement, particularly acute respiratory distress syndrome, is frequently reported, especially in pediatric patients [79, 80]. Hepatosplenic involvement is common and may manifest as hepatomegaly and splenomegaly, with imaging occasionally demonstrating diffuse periportal and pericholecystic edema [81] (Fig. 16). Neurological involvement may be severe and can occur despite normal MRI findings, reflecting underlying leptomeningitis and small-vessel vasculitis [80]. Acute kidney injury is common and multifactorial, related to hypoperfusion, increased vascular permeability, tubular injury, and thrombotic microangiopathy [80, 82].

Many patients of STy demonstrate rapid clinical improvement with appropriate antibiotic therapy alone, while the role of HLH-directed immunomodulatory treatment remains uncertain and should be individualized based on clinical severity and treatment response [80].

## Prognostic implications

Mortality in STy varies widely depending on geographic region, circulating *OT* strains, and host factors such as age, immune status, and comorbidities. A systematic review reported higher fatality rates in patients over 30 years of age, with the greatest risk observed in those above 60 years [4, 83]. Interpretation of case-fatality data is limited by marked heterogeneity across studies, ranging from national surveillance datasets to ICU-based case series. While national surveillance data from China, Japan, and South Korea report low fatality rates (~0.07–0.26%), hospital-based studies from South India report substantially higher mortality (0–33.3%) [84].

Importantly, a large systematic review demonstrated markedly higher case fatality rates in patients with specific severe organ complications identifiable on imaging, including myocarditis (42.4%), shock (39.6%), meningitis (35.5%), acute kidney injury (34.6%), acute respiratory distress syndrome (ARDS) (26.8%), hepatitis (23.2%), and thrombocytopenia (21.9%). Mortality among patients with multiorgan dysfunction syndrome (MODS) was reported to be as high as 38.9%, underscoring the prognostic value of imaging in detecting high-risk organ involvement [85].

## Management

Doxycycline is the treatment of choice for STy, typically leading to defervescence within 48 h. Early empirical use of doxycycline is crucial in endemic areas, particularly when the presentation is atypical or when the eschar is absent, to reduce the risk of complications [86]. When doxycycline is contraindicated or resistance is suspected, azithromycin is a viable alternative [87].

## Prevention

Prevention of STy focuses on personal protective measures such as wearing long-sleeved clothing, avoiding contact with vegetation, and using insect repellents. Public health education on transmission and risk reduction is essential, especially in endemic areas. Awareness among the travellers returning from endemic regions who develop fever, particularly in the presence of an eschar, to seek prompt medical attention can significantly reduce the risk of severe complications [13].

## Differential diagnosis [88–93]

**Table:** 

**Imaging feature**	**Scrub typhus**		**Leptospirosis**	**Dengue**
CNS				
Encephalitis	+		+	++
ADEM	+		+	+
Meningitis	+		+	+
Macrohemorrhage	+		+	++
Microhemorrhage	+		+	++
Infarcts	+		+	++
Cranial nerve involvement	+		+	+
Myelitis	+		+	+
Cerebellitis	+		+	+
Cytotoxic lesion of Corpus callosum (CLOCC)	+		+	++
Cerebral venous thrombosis (CVT)	Rare		Rare	Rare
Diffuse cerebral edema	+		+	+
Chest				
ARDS	++		++	++
Pneumonia	++		++	++
Pulmonary hemorrhage	+		++	++
Pleural effusion	++		++	++
Hilar and mediastinal Lymphadenopathy	+		+	+
Cardiac				
Myocarditis	+		+	++
Pericarditis	+		+	+
Pericardial effusion	+		+	++
Cardiomegaly	+		-	+
Abdomen				
GB wall thickening	++		+	++
Kidney involvement	+		+	+
Hepatomegaly	++		++	++
Splenomegaly	++		++	++
Splenic infarction	+		+	++
Splenic rupture	+		-	+
Abdominal lymphadenopathy	+		+	+
Solid organ abscess	Rare		+	-
Pancreatitis	Rare		Rare	+
Ascites	+		+	++
MSK				
Myositis	+		+	++
Reactive arthritis	+		+	++
Guillain-Barré syndrome	rare		rare	rare
Miscellaneous				
Hemorrhagic manifestations – whole body	+		+	+++
Generalised lymphadenopathy	+		+	+

## Conclusion

Scrub typhus is a significant public health concern in endemic regions, presenting with non-specific clinical features that can delay both diagnosis and treatment. Imaging is an adjunct in the diagnostic process, and helps to identify the extent of systemic involvement, guides evaluation in atypical presentations, and facilitates the monitoring of complications. With increasing accessibility to advanced imaging modalities and growing recognition of their diagnostic value, radiological assessment is becoming important in the management of scrub typhus.

## Supplementary Information

Below is the link to the electronic supplementary material.

Supplementary Material 1.

## Data Availability

No datasets were generated or analysed during the current study.
